# Pathologists in a Net-Savvy World

**DOI:** 10.4103/2153-3539.77173

**Published:** 2011-02-26

**Authors:** Rashmi Patnayak, Amitabh Jena, Amit kumar Chowhan, N. Rukamangadha, B. V. Phaneendra

**Affiliations:** Department of Pathology, India; 1 Department of Surgical Oncology, Sri Venketeswar Institute of Medical Sciences, Tirupati, Andhra Pradesh, India

Sir,

Pathology is aptly described as a bridging science as it connects the basic and clinical sciences. It also provides a link between the patients and their physicians. It helps the clinician to determine and decide different modalities of treatment (either medical or surgical) that can be provided to the patients based on the pathologist’s report. Though pathologists seldom directly interact with patients their role in the diagnosis and in providing prognostic information is invaluable. Some clinicians even pronounce the pathologists as judges and themselves as lawyers presenting a case, as pathologists provide the final diagnosis.

Computers have changed various aspects of our lives considerably since their arrival. The worldwide web of computers i.e. internet has the capability to influence various aspects of pathology like routine diagnosis, research, training and teaching.[1] Earlier, particularly in the developing countries, people used to ask pathologists whether the report is of cancer or not. But nowadays, in this era of Facebook and Twitter, to name a few, the patients come armed with all kinds of information available on the internet and they have more specific questions regarding their ailments. So what are the pathologists supposed to do to answer these ever-increasing queries?

People are well versed with different applications of computers in today’s world. Nevertheless the pathology residents should acquire the skill and get trained at the first available opportunity. Our reports also should answer the patients’ as well as the clinicians’ queries. The era of Web 1.0 is getting fast left behind with the advent of various interactive Web 2.0 social websites like Facebook and Twitter.[2,3] Following suit we need to develop Web 2.0 pathology websites whereby the educated eager patients can be provided with better answers to their queries regarding specific tests, diseases, laboratory reports and prognosis. In view of this we can create relevant, authentic and well-updated links to laboratory test results, thereby minimizing the volume of ancillary text presented.[4] Since the cyber world is a goldmine for data storage, its potential can be explored to achieve a healthy green globe. Simultaneously, one should also provide judicious information keeping in mind the probable legal aspect.

Care should be taken to provide separate icons for the patients’ and clinicians’ query as the clinicians would be interested in more in-depth analysis which can be addressed with the use of medical terminology, which patients may not be able to grasp. Furthermore, the patients will be more interested in the prognostic part, which needs to be explained in layman’s words. One way to achieve this is to develop FAQs (Frequently Asked Questions) section with general questions and answers mostly asked by patients. In this regard an article by Epstein JI, “The FAQ Initiative Explaining Pathology Reports to Patients” raises several relevant points.[5] We fully agree with the author that definitely pathologists should take part actively in patient care in days to come. What we feel is gradually we have to expand our FAQs (Frequently Asked Questions) so as to include cases other than malignant ones. Anyway this is a welcome initiative. Definitely with the FAQ initiative the patients will become active participants in their own healthcare and this need not necessarily interfere with the relationship between the patient, clinicians and pathologists.[6]
